# Clinical application and effectiveness analysis of modified Asia–Pacific colorectal cancer screening score combined with fecal occult blood test for colorectal cancer screening in asymptomatic population

**DOI:** 10.1186/s12876-025-03779-1

**Published:** 2025-03-31

**Authors:** Lin Li, Tianzheng Wang, Chiyi He, Xiaoping Niu

**Affiliations:** Departments of Gastroenterology, Yijishan Hospital (The First Affiliated Hospital of Wannan Medical College), Wuhu, 241001 P.R. China

**Keywords:** Colorectal cancer, Screening and diagnosis, Early detection, Tumor

## Abstract

**Aims:**

To evaluate the value of the modified Asia–Pacific colorectal cancer screening (APCS) scoring system combined with fecal occult blood test (FOBT) for colorectal cancer (CRC) screening in asymptomatic population of Wannan region.

**Methods:**

We prospectively collected and investigated asymptomatic individuals attending Yijishan Hospital (the first affiliated hospital of Wannan Medical College) from January 1, 2021 to December 31, 2022. All enrolled patients received modified APCS scores and FOBT before colonoscopy. We used receiver operating characteristic (ROC) curves to estimate the screening value of the modified APCS score combined with FOBT. We also retrospectively collected patients with stage T1 CRC to explore the independent risk factors for lymph node metastasis (LNM) of CRC.

**Results:**

513 participants were finally included in the study. The combined detection of modified APCS score and FOBT can be divided into 5 groups [modified APCS high risk &FOBT + (T1 group), modified APCS high risk &FOBT- (T2 group), modified APCS medium risk &FOBT + (T3 group), modified APCS medium risk &FOBT- (T4 group), and modified APCS low risk &FOBT- (T5 group)], the detection rates of CRC were 9.09%, 1.67%, 5.77%, 0.92% and 0%, respectively. The detection rate of advanced adenoma was 25.76%, 35.00%, 21.15%, 2.75% and 1.96%, respectively. The detection rate of CRC in T1 group was 9.88 times that in T4 group, and the detection rate of advanced adenoma was 9.36 times that in T4 group. The high-risk group and positive rate of modified APCS were indicators for further colonoscopy. Tumor gross morphology, tumor differentiation degree, and nerve infiltration were independent risk factors for T1 CRC LNM.

**Conclusions:**

The combination of modified APCS score and FOBT test has important clinical application value in the preliminary screening of colorectal tumors in asymptomatic population. For selected T1 CRC patients, if the lesion is ulcerative and the pathology indicates low differentiation, endoscopic submucosal dissection (ESD) treatment should be carefully selected to prevent the risk of LNM.

**Supplementary Information:**

The online version contains supplementary material available at 10.1186/s12876-025-03779-1.

## Introduction

Colorectal cancer (CRC) is the third most common cancer in China and the fifth most common cancer in mortality. In Asia, the incidence and mortality of CRC are rising rapidly, and in China, CRC is showing rapid growth in cities, which is higher than that in rural areas [1]. According to statistics, there were 388,000 new cases of CRC in China in 2015, with a rapid increase of 7.4% per year, and by 2020 there were 555,000 new cases of CRC in China; The death rate of CRC in China increased from 5.39% to 7.23% between 2004 and 2018, with 286,000 deaths [2, 3]. The most common pathway for CRC development is progression through multiple stages, hyperplasia–small adenoma–large adenoma–severe atypical hyperplasia–early adenocarcinoma–advanced adenocarcinoma. There is strong evidence that screening for CRC improves survival [4]. Wannan region of Anhui Province is one of the regions with high incidence of CRC in China. The incidence of CRC was on the rise, and the rural–urban differences, age distribution, and gender differences are consistent with national trends, but screening rates and early diagnosis rates may be lower. In response to the Healthy China 2030 plan, it is necessary to strengthen the early screening of CRC in Wannan region and explore the risk factors of CRC.

At present, the screening mode for CRC in China is still to conduct colonoscopy after the initial screening of positive fecal occult blood test (FOBT), and clinical risk assessment has not been included in the initial screening method. However, due to the limited resources of colonoscopy in China, colonoscopy of all positive patients cannot be completed in a short time. The target population can be stratified by CRC clinical risk score, which facilitates reasonable allocation of colonoscopy resources and control screening costs.

The Asia Pacific Colorectal Screening Score (APCS) is a questionnaire derived from a prospective, multicenter, cross-sectional clinical study conducted in 11 countries in Asia [5]. It is an effective tool for opportunistic screening of asymptomatic CRC in the Asia–Pacific population, including four variables: age, sex, family history of CRC, and smoking [6]. In recent years, a modified APCS scoring system has been developed, comprising demographic risk factors: gender, age, body mass index (BMI), smoking history, and family history of CRC. Total scores of 0, 1–2, and 3–6 were defined as low, medium, and high risk, respectively [7]. It is more suitable for people in the Asia–Pacific region, especially for those with high risk factors such as obesity and diabetes.

T1 CRC is defined as cancer with a depth of invasion limited to the mucosa or submucosa, regardless of lymph node metastasis (LNM) [8]. Patients with T1 CRC underwent endoscopic submucosal dissection (ESD), with a 5-year survival rate of more than 95% and a favorable prognosis [9]. The risk of LNM in T1 CRC confined to the intramucosal layer (T1a) is small, while the rate of LNM in submucosal carcinoma (T1b) is about 10% [10]. CRC infiltrating into the submucosa is still at risk of LNM after ESD treatment, but the risk factors for LNM in T1 CRC are not yet clear.

To date, there are few studies on the application of the modified APCS scoring system combined with FOBT to the opportunistic screening of asymptomatic physical examination population. The study aims to evaluate the effectiveness of modified APCS combined with FOBT for CRC screening in asymptomatic population of Wannan region, which can provide a new idea for our existing screening methods for CRC. Moreover, the risk factors for LNM in T1 CRC patients from the Wannan region were further analyzed.

## Patients and methods

### Prospective study population

We prospectively collected and investigated asymptomatic individuals attending physical examination center of Yijishan Hospital, Wannan Medical College, Anhui Province, China from January 1, 2021 to December 31, 2022. All study participants volunteered to participate in the study and signed an informed consent form. Modified APCS questionnaire score and FOBT in participants aged ≥ 40 years were measured. We excluded participants as follows: (1) patients with confirmed or highly suspected CRC; (2) those who have been diagnosed with colorectal polyps to be resected; (3) patients with warning symptoms of CRC: blood in the stool, anemia, changes in bowel habits or unexplained weight loss in the past 3 months; (4) history of malignant tumor; (5) participants with missing or incomplete data.

### Modified APCS scoring system

As shown in Table 1, scores 0, 1 to 2, and 3 to 6 were combined to form three categories, corresponding to groups with a low, medium, and high risk of CRC, respectively.

**Table 1:** General data of enrolled patients and univariate analysis of modified APCS scores

Variables	Score	n (%)	Colorectal tumors		χ2	P
			Yes (*n* = 169)	No (*n* = 344)		
Age					75.977	< 0.001
40–49	0	240 (46.78)	45	195		
50–59	1	176 (34.31)	58	118		
≥ 60	2	97 (18.91)	66	31		
Gender					9.734	0.002
Male	1	290 (56.53)	112	178		
Female	0	223 (43.47)	57	166		
BMI (kg/m^2^)					8.397	0.004
< 23.9	0	353 (68.81)	102	251		
≥ 23.9	1	160 (31.19)	67	93		
Smoking history					36.923	< 0.001
Current or past	1	196 (38.21)	96	100		
Never	0	317 (61.79)	73	244		
Family history of CRC					2.457	0.117
Present	1	18 (3.51)	9	9		
Absent	0	495 (96.49)	160	335		

*Abbreviations:* *APCS* Asia Pacific Colorectal Screening Score, *CRC* colorectal cancer, *BMI* body mass index

### FOBT

**Fig. 1:**
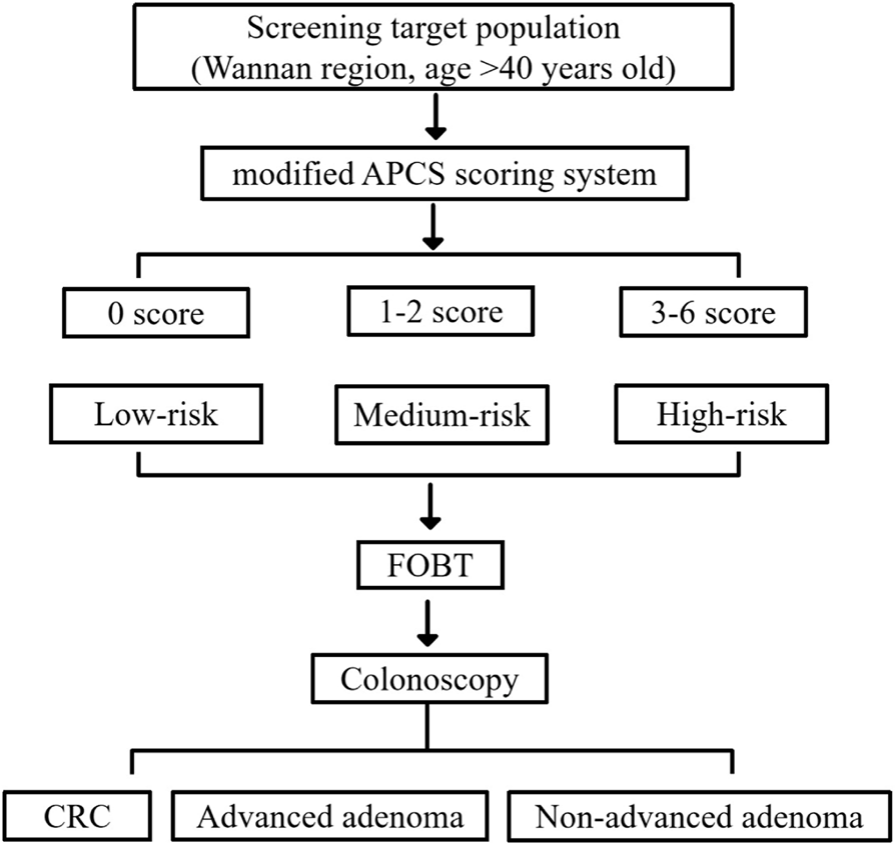
Screening process of early-stage CRC in Wannan region. Abbreviations: APCS: Asia Pacific Colorectal Screening Score; CRC: colorectal cancer; FOBT: fecal occult blood test

Before undergoing coloscopy, fecal specimens were collected under the guidance of special personnel, and fecal occulted blood detection kit (tetramethylbenzidine chemical method combined with immunocolloidal gold method/double method) was used for detection. Fecal hemoglobin exceeding 2 μg/mL (chemical method) or 0.2 μg/mL (immunomethod) was defined as FOBT-positive. For patients with positive FOBT are recommended to complete colonoscopy. The early CRC screening process is shown in Fig. 1.

### Colonoscopy

Colonoscopy and pathology results were confirmed by experienced endoscopists (physicians with more than 3 years of experience in colonoscopy operation, and the duration of endoscopic removal ≥ 6 min)) and pathologists (each specimen was independently reviewed by 2 physicians with professional titles or above of attending physicians), respectively. Colorectal tumors are classified into CRC, advanced colorectal adenomas and non-advanced colorectal adenomas. Advanced colorectal adenoma is diagnosed with one or more of the following: ① the maximum diameter of the tumor is > 10 mm; ② The tissue contains villous structure; ③ high-grade intraepithelial neoplasia or severe dysplasia [9]. The pathological results were adenomas, but those that did not have the above characteristics of advanced colorectal adenomas were non-advanced colorectal adenomas. The gold standard for CRC is pathological findings.

### Retrospective study population

Patients who received radical resection of CRC in Yijishan Hospital, Wannan Medical College from January 2010 to December 2022 and proved to be stage T1 by postoperative pathology were selected. Exclusion criteria: (1) patients with surgical recurrence or heterochronous tumor; (2) patients with familial adenomatous polyposis or hereditary non-polyposis CRC; (3) patients with incomplete pathological data.

Clinicopathological data of enrolled patients were collected, including: gender, age, tumor site, tumor length diameter, circumference of intestinal wall involved, gross classification, degree of pathological differentiation, and depth of invasion (T1a: mucosa; T1b: submucosa), nerve invasion, vascular invasion, number of lymph nodes dissected, and LNM were analyzed to determine the correlation between the above factors and early CRC LNM.

### Statistical analysis

We used IBM SPSS26.0 (IBM Corp., Armonk, NY, USA) to analyze the data. Measurement data are presented as mean ± standard deviation (SD), and counting data are presented as number (%). Chi-square test, Fisher's exact test or continuity correction test were used for comparison between groups, *P* < 0.05 was statistically significant. Bonferroni correction method was used for pairwise comparison among the three groups, *P* < 0.016 indicates a statistically significant difference. Receiver operating characteristic (ROC) curve was used to determine the diagnostic value of the modified APCS score combined with FOBT in CRC. Additionally, Chi-square test or Fisher's exact test were used for counting data groups. Binary Logistic regression analysis model was used for multivariate analysis.* P* < 0.05 was statistically significant.

## Results

### Patient characteristics

**Fig. 2:**
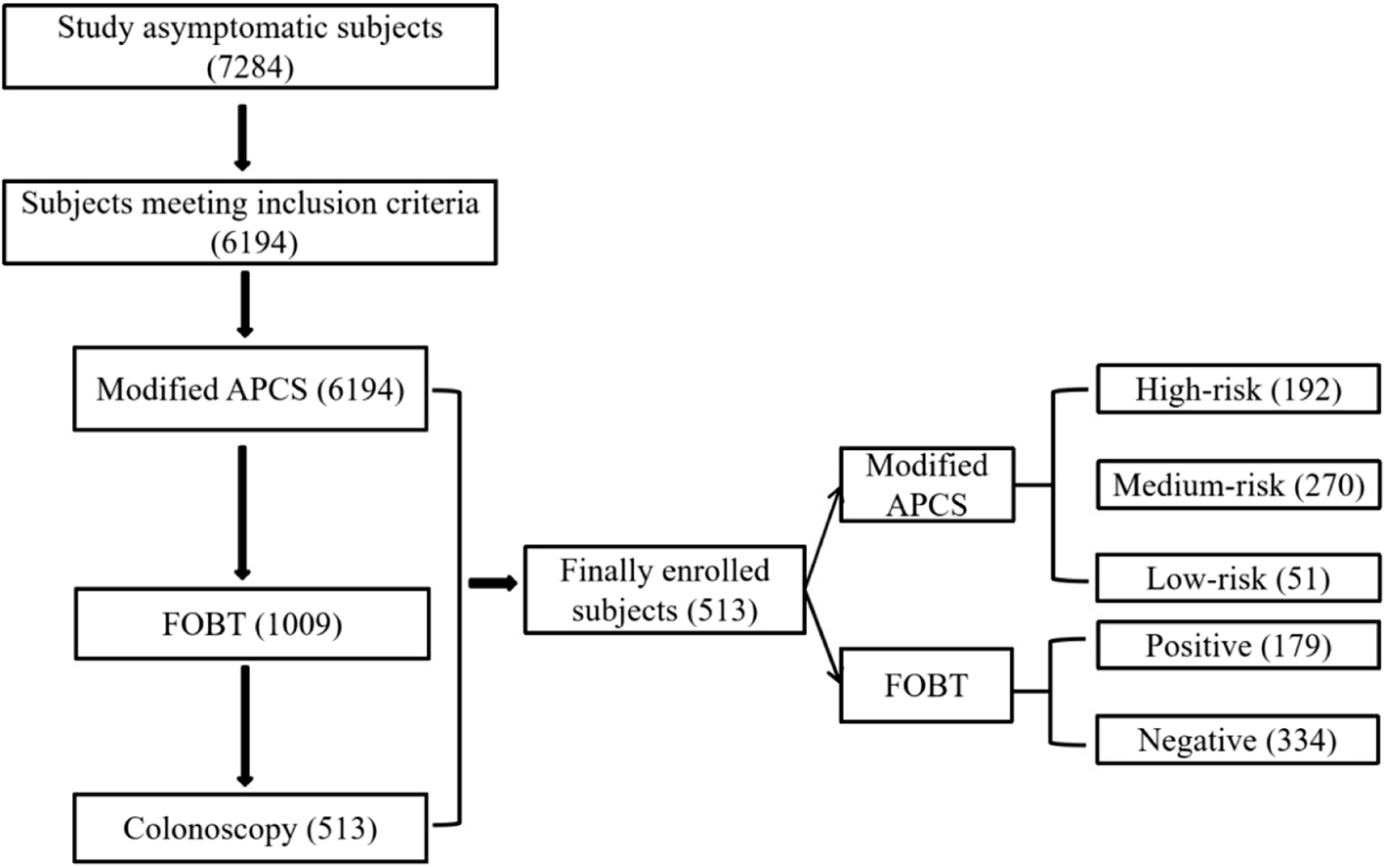
Asymptomatic CRC opportunistic screening population inclusion and risk stratification process. Abbreviations: APCS: Asia Pacific Colorectal Screening Score; CRC: colorectal cancer; FOBT: fecal occult blood test

A total of 7284 participants underwent opportunistic CRC screening, 6194 met the study inclusion criteria and completed the modified APCS questionnaire, 1009 (16.29%) completed FOBT, and 513 (8.28%) completed colonoscopy. Therefore, 513 (8.28%) participants completed the modified APCS questionnaire, FOBT and colonoscopy at the same time, and were finally included in the further analysis of the study (Fig. 2).

As shown in Table 1, there were 240 people aged 40–49 years (46.78%), 176 people aged 50–59 years (34.31%), 97 people aged ≥ 60 years (18.91%); 290 males (56.53%) and 223 (43.47%) females; 353 (68.81%) and 160 (31.19%) patients with BMI < 23.9kg/m^2^ and ≥ 23.9kg/m^2^, respectively; 196 cases (38.21%) had smoking history, while 317 (61.79%) cases had no smoking history; 18 cases (3.51%) had a family history of CRC in a first-degree relative. Colorectal tumors were detected in 169 (32.94%) cases including cancer in 18 (3.51%) cases, advanced colorectal adenomas in 73 (14.23%) cases, and non-advanced colorectal adenomas in 78 (15.20%) cases.

### Efficacy of the modified APCS scoring system in CRC screening

Firstly, as shown in Table 1, we performed a univariate analysis of the modified APCS scoring system to screen for CRC risk factors, and the results suggested that the variables "gender, age, smoking history, BMI" were associated with CRC development, and have a family history of CRC was not associated with CRC development in this study.

Secondly, by using the modified APCS scoring system, 192 cases (37.43%) in high-risk group, 270 cases (52.63%) in medium-risk group and 51 cases (9.94%) in low-risk group were screened out (Table 2).

1. CRC was detected in 13 cases in the high-risk group with a detection rate of 6.77%, and 5 cases in the medium-risk group with a detection rate of 1.85%. However, in the low-risk group, 0 cases of CRC were detected. The detection rate of high-risk group was 3.66 times that of medium-risk group. The difference between groups was statistically significant (χ2 = 8.71,* P* = 0.01).
2. 55 cases of advanced colorectal adenoma were detected in the high-risk group, with a detection rate of 28.65%; 17 cases were detected in the medium-risk group, with a detection rate of 6.30%; and 1 case was detected in the low-risk group, with a detection rate of 1.96%. The detection rate of high-risk group was 4.55 times that of medium-risk group and 14.62 times that of low-risk group. The detection rate of the medium-risk group was 3.21 times that of the low-risk group. The difference between groups was statistically significant (χ2 = 52.91, *P* < 0.001).
3. 47 cases of non-advanced colorectal adenoma were detected in the high-risk group, with a detection rate of 24.48%, 29 cases in the medium-risk group with a detection rate of 10.74%, and 2 cases in the low-risk group with a detection rate of 3.92%. The detection rate of high-risk group was 2.28 times that of medium-risk group and 6.24 times that of low-risk group. The detection rate of the medium-risk group was 2.74 times that of the low-risk group. The difference between groups was statistically significant (χ2 = 22.02, *P* < 0.001).

**Table 2:** Efficacy of the modified APCS scoring system, FOBT and modified APCS scoring system combined with FOBT in CRC screening

Item	n	CRC (*n* = 18)			Advanced colorectal adenoma (*n* = 73)			Non-advanced colorectal adenoma (*n* = 78)		
		n (%)	χ2	P	n (%)	χ2	P	n (%)	χ2	P
modified APCS			8.71	0.01		52.91	< 0.001		22.02	< 0.001
high-risk	192	13 (6.77)			55 (28.65)			47 (24.48)		
medium- risk	270	5 (1.85)			17 (6.30)			29 (10.74)		
low-risk	51	0			1 (1.96)			2 (3.92)		
FOBT			19.27	< 0.001		14.84	< 0.001		0.95	0.329
positive	179	15 (8.38)			40 (22.35)			31 (17.32)		
negative	334	3 (0.90)			33 (9.88)			47 (14.07)		
Combined			16.92	0.001		67.44	< 0.001		31.78	< 0.001
T1	132	12 (9.09)			34 (25.76)			31 (23.48)		
T2	60	1 (1.67)			21 (35.00)			16 (26.67)		
T3	52	3 (5.77)			11 (21.15)			0		
T4	218	2 (0.92)			6 (2.75)			29 (13.30)		
T5	51	0			1 (1.96)			2 (3.92)		

*Abbreviations* *APCS* Asia Pacific Colorectal Screening Score, *CRC* colorectal cancer, *FOBT* fecal occult blood test, *T1 *modified APCS high risk &FOBT + , *T2* modified APCS high risk &FOBT-, *T3* modified APCS medium risk &FOBT +, *T4* modified APCS medium risk &FOBT-, *T5* modified APCS low risk &FOBT-

### Efficacy of FOBT in CRC screening

In this study, 179 cases (34.89%) were positive for FOBT and 334 cases (65.10%) were negative (Table 2).

1. CRC was detected in 15 (8.38%) cases in the FOBT positive group and 3 (0.90%) cases in the OB negative group. The detection rate of FOBT positive group was 9.31 times that of FOBT negative group. The difference between groups was statistically significant (χ2 = 19.27, *P* < 0.001).
2. 40 cases of advanced colorectal adenoma were detected in FOBT positive group, with a detection rate of 22.35%, and 33 cases were detected in FOBT negative group, with a detection rate of 9.88%. The detection rate of FOBT positive group was 2.26 times that of FOBT negative group. The difference between groups was statistically significant (χ2 = 14.84, *P* < 0.001).
3. 31 cases of non-advanced colorectal adenoma were detected in FOBT positive group, with a detection rate of 17.32%, and 16 cases were detected in FOBT negative group, with a detection rate of 14.07%. There was no significant difference in the detection of non-advanced colorectal adenomas between the two groups (χ2 = 0.95, *P* = 0.329).

### Efficacy of the modified APCS scoring system combined with FOBT in CRC screening

We divided the enrolled subjects into the following 5 groups: modified APCS high risk &FOBT + (T1 group), modified APCS high risk &FOBT- (T2 group), modified APCS medium risk &FOBT + (T3 group), modified APCS medium risk &FOBT- (T4 group), and modified APCS low risk &FOBT- (T5 group) (Table 2).

1. 132 patients were in T1 group, CRC, advanced colorectal adenoma and non-advanced colorectal adenoma were screened out in 12 (9.09%), 34 (25.76%) and 31 (23.48%) cases, respectively.
2. 60 patients were in T2 group, CRC, advanced colorectal adenoma, non-advanced adenoma were detected in 1 (1.67%), 21 (35.00%), and 16 (26.67%) cases, respectively.
3. 52 cases were in T3 group, of which 3 (5.77%) cases of CRC were detected, and 11 (21.15%) cases of advanced colorectal adenoma were screened out.
4. 218 cases were in T4 group, 2 (0.92%) cases of CRC, 6 (2.75%) cases of advanced colorectal adenoma and 29 (13.30%) cases of non-advanced colorectal adenoma were detected, respectively.
5. 51 patients were in T5 group, advanced colorectal adenoma and non-advanced colorectal adenoma were detected in 1 (1.96%), 2 (3.92%) cases, respectively.

The CRC detection rate, advanced adenoma detection rate and non-advanced adenoma detection rate of T1 group were 9.88 times, 9.36 times and 1.77 times of T4 group, respectively.The detection rate of CRC in T3 group was 3.46 times higher than that in T2 group, and the detection rate of advanced adenoma in T2 group was 1.65 times higher than that in T3 group.

### ROC curves for diagnostic cutoffs of modified APCS score combined with FOBT in CRC

As shown in Table 3, in the screening of CRC, the sensitivity and negative predictive value of modified APCS score combined with FOBT screening (88.89%, 99.26%) were higher than those of modified APCS score (72.22%, 98.44%) and FOBT alone (83.33%, 99.10%) respectively. However, the specificity of combined screening (53.93%) was lower than that of FOBT alone (66.87%) and modified APCS score alone (63.84%). In the screening of advanced colorectal adenoma, the sensitivity and negative predictive value of modified APCS score combined with FOBT screening (90.11%, 96.65%) were higher than those of modified APCS score (74.73%, 92.79%) and FOBT alone (60.44%, 89.22%) respectively. However, the specificity of combined screening (61.66%) was lower than FOBT alone (70.62%) and modified APCS score alone (70.62%).

**Table 3:** Sensitivity, specificity, positive predictive value and negative predictive value of three screening methods

Item	Screening methods	CRC	Advanced colorectal adenoma
Sensitivity(%) (95%CI)	modified APCS	72.22 (46.52–90.31)	74.73 (64.53–83.25)
	FOBT	83.33 (58.58–96.42)	60.44 (49.64–70.54)
	Combined	88.89 (65.29–98.63)	90.11 (82.059–5.377)
Specificity(%)(95%CI)	modified APCS	63.84 (59.43–68.08)	70.62 (66.02–74.92)
	FOBT	66.87 (62.53–71.01)	70.62 (66.02–74.92)
	Combined	53.94 (49.44–58.40)	61.61 (56.79–66.27)
Positive predictive value(%) (95%CI)	modified APCS	6.77 (5.06–9.01)	35.42 (31.20–39.88)
	FOBT	8.38 (6.70–10.43)	30.73 (26.20–35.65)
	Combined	6.56 (5.49–7.82)	33.61 (30.59–36.77)
Negative predictive value(%) (95%CI)	modified APCS	98.44 (96.77–99.26)	92.84 (90.05–94.88)
	FOBT	99.10 (97.51–99.68)	89.22 (86.44–91.49)
	Combined	99.26 (97.30–99.80)	96.65 (93.93–98.18)

*Abbreviations:* *APCS* Asia Pacific Colorectal Screening Score, *CRC* colorectal cancer, *FOBT* fecal occult blood test, *CI* confidence interval

**Fig. 3:**
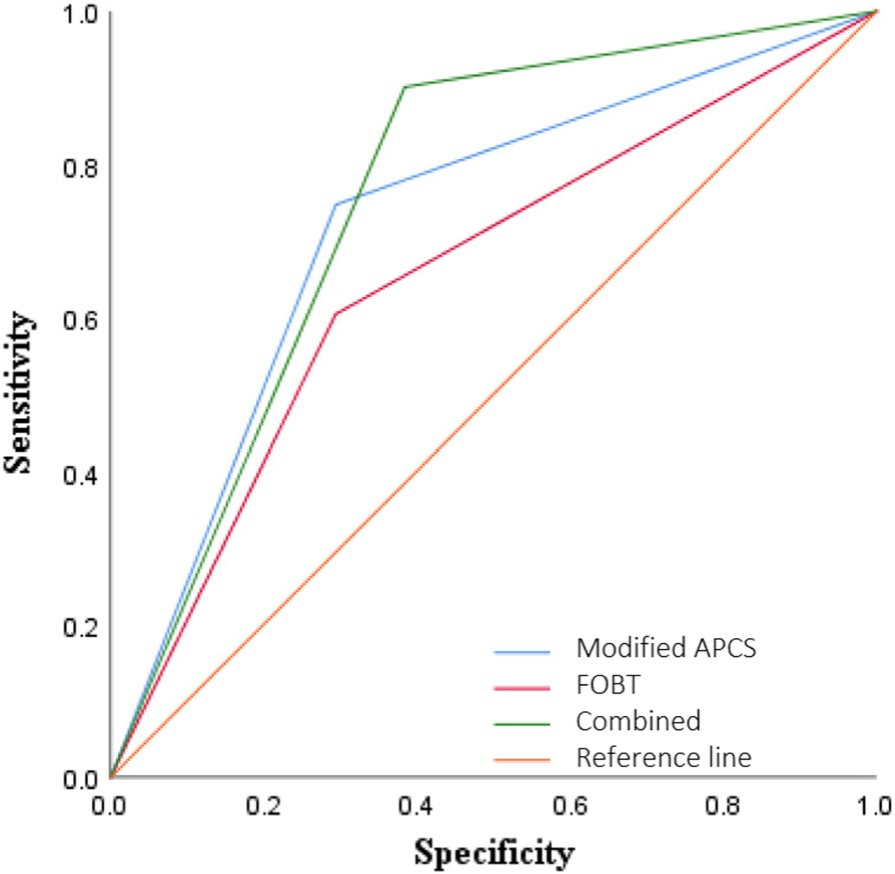
Receiver operating characteristic (ROC) curves for diagnosing CRC. Abbreviations: ROC: receiver operating characteristic; APCS: Asia Pacific Colorectal Screening Score; FOBT, fecal occult blood test; CRC: colorectal cancer. ROC curve of combined is means the combination of modified APCS score, and FOBT for diagnosing CRC

Furthermore, ROC curves were performed to estimate the value of modified APCS score combined with FOBT in screening for CRC. CRC and advanced adenoma were selected as case group (*n* = 91), and non-advanced adenoma, other benign lesions and normal colon were selected as control group (*n* = 422). As shown in Table 4, the sensitivity (90.11%) and negative predictive value (96.65%) of the combination of modified APCS score and FOBT were higher than those of modified APCS score alone (74.73%, 92.84%) and FOBT screening alone (60.44%, 89.22%). In addition, ROC curves of different screening methods for advanced tumors were plotted, as shown in Fig. 3, modified APCS score and FOBT were both of diagnostic value for CRC. The area under the curve (AUC) of modified APCS score combined with FOBT (AUC: 0.759, 95% confidence interval [CI]: 0.710–0.807, *P* < 0.001) were higher than FOBT (AUC: 0.655, 95%CI: 0.592–0.719, *P* < 0.001) and APCS scores (AUC: 0.727, 95%CI: 0.727), respectively, indicating that the combination of modified APCS score combined with FOBT was more effective in screening advanced tumors (Table 4).

**Table 4:** The accuracy of different screening methods in diagnosing CRC

Screening methods	Sensitivity (%)	Specificity (%)	Positive predictive value (%)	Negative predictive value (%)	AUC	P	95%CI
Modified APCS	74.73 (64.527–83.254)	70.62 (66.018–74.923)	35.42 (31.197–39.876)	92.84 (90.052–94.884)	0.727	< 0.001	0.669 ~ 0.784
FOBT	60.44 (49.640–70.540)	72.97 (67.942–77.588)	30.73 (26.203–35.653)	89.22 (86.439–91.489)	0.655	< 0.001	0.592 ~ 0.719
Combined	90.11 (82.054–95.377%)	61.61 (56.785–66.274)	33.61 (30.585–36.769)	96.65 (93.927–98.180)	0.759	< 0.001	0.710 ~ 0.807

*Abbreviations:* *APCS* Asia Pacific Colorectal Screening Score, *CRC* colorectal cancer, *FOBT* fecal occult blood test, *AUC* area under the receiver operating characteristic curve, *CI* confidence interval

### Multivariate analysis of lymph node metastasis in T1 CRC patients

When treating patients with T1 CRC with ESD, the risk of T1 CRC LNM should be evaluated. Additional colectomy should be considered if there is an increased risk of LNM. Therefore, risk factors for LNM in T1 CRC patients were analyzed.

A total of 155 patients were enrolled in this study and divided into LNM group and non-LNM group. The results showed that the tumor gross morphology, tumor differentiation degree and nerve infiltration were correlated with LNM in T1 CRC patients (*P* < 0.05, Table 5).

**Table 5:** Univariate analysis of lymph node metastasis in T1 CRC patients

Risk factors	LNM + (*n* = 19)	LNM- (*n* = 136)	χ^2^	P
Gender			0.523	0.474
Male (*n* = 86)	12	74		
Female (*n* = 69)	7	62		
Age			0.614	0.454
< 55 (*n* = 35)	3	32		
≥ 55 (*n* = 120)	16	104		
Location				0.689^*^
Right semicolon (*n* = 16)	1	15		
Left semicolon (*n* = 40)	6	34		
Rectum (*n* = 99)	12	87		
Size			1.293	0.252
≥ 2cm (*n* = 77)	9	68		
< 2cm (*n* = 78)	10	68		
Gross morphology			12.543	< 0.001
Elevated (*n* = 133)	12	121		
Ulcerative (*n* = 22)	7	15		
Differentiation degree			11.183	< 0.001
Poorly (*n* = 9)	4	5		
Medium–high (*n* = 146)	15	131		
Depth of infiltration				0.674^*^
T1a (*n* = 16)	1	15		
T1b (*n* = 139)	18	121		
Involute intestinal diameter			0.431	0.525
< 1/4 (*n* = 34)	3	31		
≥ 1/4 (*n* = 121)	16	105		
Nerve infiltration				< 0.001^*^
Presence (*n* = 5)	4	1		
Absence (*n* = 150)	15	135		
Vascular infiltration			0.009	0.924
Presence (*n* = 17)	7	10		
Absence (*n* = 138)	12	126		

*Abbreviations:* *CRC* colorectal cancer, *LNM* lymph node metastasis

We further conducted a multivariate analysis of the risk factors for LNM in T1 CRC patients. The results showed that tumor gross morphology (odds ratio [OR], 2.939; 95% CI, 1.173–7.363; *P* = 0.021), tumor differentiation degree (OR, 6.562; 95% CI, 3.149–25.687; *P* < 0.001), and nerve infiltration (OR, 32.585; 95% CI, 3.288–322.949; *P* < 0.001) were independent risk factors for T1 CRC LNM (Table 6).

**Table 6:** Multivariate analysis of lymph node metastasis in T1 CRC patients

Risk factors	B	S.E	Wald	*P*	OR	95%CI
Gross morphology	1.078	0.469	5.289	0.021	2.939	1.173–7.363
Differentiation degree	2.197	0.535	16.829	< 0.001	6.562	3.149–25.687
Nerve infiltration	3.484	1.170	8.863	0.003	32.585	3.288–322.949
Constant	−10.856	2.659	16.670	< 0.001		

*Abbreviations:* *CRC* colorectal cancer, *OR* odds ratio, *CI* confidence interval

## Discussion

At present, the occurrence of most CRC is in line with the recognized " hyperplasia–small adenoma–large adenoma–severe atypical hyperplasia–early adenocarcinoma–advanced adenocarcinoma." theory, and this process can experience more than 10 years [11]. The detection rate of adenoma in asymptomatic population is reported to be 12.5%−36% [12], so early screening of colorectal adenoma in this window period is an important means to reduce the mortality of CRC, and it is also the focus of prevention and treatment of CRC. Another review found that access to screening colonoscopy (2 studies, *n* = 436,927) or fecal immunochemical testing (FIT, 1 study, *n* = 5.4 million) was associated with a lower risk of CRC morbidity or mortality when compared with those who were not screened [13]. In the West, colonoscopy-based screening programs have placed a huge burden on health care systems. This study was the first to apply modified APCS combined with FOBT to screen CRC in asymptomatic populations in Wannan region, and the results suggested that the use of modified APCS combined with FOBT was a convincing and feasible protocol for screening CRC.

The APCS was originally developed by experts in the Asia–Pacific region to assess CRC risk, and the following four independent risk factors were identified as the basis for risk stratification: age (2 points for 50 to 69 years, 3 points for 70 years and older), gender (1 point for men and 0 points for women), family history (2 points for first-degree relatives with CRC, 0 points for those without), and smoking history (1 point for smokers, 0 score for non-smokers). The risk levels of the subjects were divided into three levels: low risk group (0–1 points), medium risk group (2–3 points) and high risk group (4–7 points) [5, 14]. However, the modified APCS adjusted age groups and score weights on the basis of APCS, adjusting the age group score to 1 point for 50–59 years old, 2 points for ≥ 60 years old, and adjusting the score of first-degree relatives with CRC from 2 points to 1 point. BMI was added as a risk factor (0 for < 23 kg/m^2^ and 1 for ≥ 23 kg/m^2^). Modified APCS is more suitable for people in the Asia–Pacific region, especially for those with high risk factors such as obesity and diabetes. By adjusting the scoring criteria, it aims to improve the accuracy of CRC risk assessment.

A multicenter study initially explored the use of APCS scores in conjunction with quantitative fecal immunochemical test (QFIT) tests in recommending the need for colonoscopy in asymptomatic populations. The results suggest that the algorithm based on APCS score can greatly reduce the workload of colonoscopy when screening subjects for FIT or colonoscopy [6]. Some scholars have expanded the application of APCS score. For example, Wang et al*.* [15] verified the application of APCS score in symptomatic patients and found that APCS score combined with metabolic syndrome and obesity had a good effect on screening advanced tumors. Zhang et al. [16] proposed a modified APCS score based on the APCS score, which included body mass index and the number of patients with family history into the evaluation criteria, and proved that it also had a good effect on screening for advanced tumors. One study [17] comparing the efficacy of seven scores in screening for advanced tumors found that the sensitivity of the modified APCS score was 39% (95%CI: 34%−44%) and the specificity was 81% (95%CI: 80%−82%), however the sensitivity of the modified APCS score in screening for CRC in asymptomatic people was lower than that in our study.

We first analyzed the screening efficiency of each risk factor in the modified APCS score, and the results suggested that age, gender, BMI, and smoking history had statistical significance in the detection of colorectal tumors (*P* < 0. 05), there was no statistically significant difference in family history of CRC among first-degree relatives, which was considered to be related to the small sample size. In addition, confounding factors such as age, sex, diet, lifestyle, and so on can mask the true impact of family history. Therefore large sample sizes, multi-center studies, and long-term follow-up are needed to more accurately assess the association between family history and CRC.

In our study, the proportion of modified APCS score associated with CRC risk was 6.77% in the high-risk group, 1.85% in the medium-risk group, and 0% in the low-risk group. Only 1 patient (1.96%) in the low-risk group having advanced adenoma risk. Screening only high- and medium-risk patients using the modified APCS score prevented 50 low-risk patients from undergoing colonoscopy. The risk of CRC was associated with 8.38% in the FOBT-positive group and 0.9% in the negative group. Low adherence to FOBT limits its benefit for CRC screening. It is expected to improve the compliance of patients using FOBT by increasing the publicity of the application of non-invasive early screening technology to CRC screening in large populations, especially in community or primary hospitals, and using multimedia for popular science publicity.

Compared with modified APCS score alone and FOBT alone, modified APCS combined with FOBT had a higher CRC detection rate (9.09%), improving diagnostic capacity for CRC. The detection rate of CRC in modified APCS combined with FOBT group was T1 > T3 > T2 > T4 (χ2 = 16.92, *P* = 0.001). On the other hand, the detection rate of advanced adenoma was T2 > T1 > T3 > T4 > T5 (χ2 = 67.44, *P* < 0.001), and the detection rate of non-advanced adenoma was T2 > T1 > T4 > T5 (χ2 = 31.78, *P* < 0.001). Therefore, colonoscopy is recommended for patients with positive FOBT results and modified APCS score as high risk, followed by patients with positive FOBT results and modified APCS score as medium risk.

However, challenges remain regarding the implementation of this approach in the real world. The modified APCS scoring only needs to collect basic information without laboratory testing or expensive equipment. Through risk stratification, high-risk groups can be screened preferentially and over-screening of low-risk groups can be reduced, which has the advantages of low cost and high efficiency. However, questionnaire surveys are required, which is a huge workload for CRC screening in a large population. Fecal occult blood test is a widely used screening tool with high sensitivity, specificity and low cost, especially in resource-limited areas, but the willingness of the population to cooperate with fecal testing needs to be further improved. Colonoscopy screening programs require specialized equipment, endoscopists and anesthesia support, are expensive, and may be difficult to scale up in areas with limited resources, and are more suitable as a diagnostic tool for high-risk populations. Therefore, phased implementation is recommended: in resource-limited areas, modified APCS score and FOBT can be promoted first, and colonoscopy can be gradually introduced. On the other hand, the training of primary medical staff should be strengthened to improve the implementation capacity of FOBT and modified APCS scoring. Establish a referral mechanism to ensure that positive cases receive colonoscopy in a timely manner. Governments should provide financial support to reduce screening costs and increase coverage, while promoting public education and increasing screening participation.

When a diagnosis of T1 CRC or advanced adenoma through CRC screening requires ESD treatment, we should assess the risk of T1 CRC LNM. The predictors of T1 CRC LNM have not been uniformly determined. A recent study found that left-sided CRC, deep submucosal invasion depth, poor histological grade, lymphatic invasion, venous invasion and tumor budding grade 2/3 were significant risk factors for LNM [18]. The results of our study were consistent with these findings, suggesting that the degree of tumor differentiation (including low differentiation) and nerve invasion were significantly correlated with LNM, and were independent risk factors for T1 CRC LNM. In addition, our study also found that CRC with a gross classification of ulcerative type was an independent risk factor for LNM. Generally speaking, the lower the degree of differentiation, the higher the degree of malignancy and the worse the prognosis. In this study, location and vascular infiltration were not found to be a risk factor for LNM in T1 CRC patients, which may be related to the small sample size. Therefore, for T1 CRC with low differentiation and ulcerative type indicated by preoperative pathology, endoscopic ultrasonography, enhanced CT and other methods should be further evaluated for LNM before treatment to determine whether direct radical surgical treatment is required. Or for patients who have undergone ESD treatment, additional surgery should be considered if the post-ESD pathology suggests ulcerative, poorly differentiated tumors and/or nerve infiltration.

There are some limitations in this study. First of all, this study is a single-center study with a small amount of data. Secondly, the inclusion and exclusion criteria of this study were strictly formulated, and the included subjects were asymptomatic subjects as much as possible. However, since the contents of the questionnaire were all provided by the subjects themselves, errors caused by memory factors were unavoidable. Thirdly, compared with traditional APCS, the modified APCS may perform better in some high-risk populations, but the benefit in low-risk populations may be limited, which means that we need to further refine the APCS score by including more risk factors and evaluate its effect in combination with FOBT in screening for advanced colorectal tumors. Finally, participants were not followed up. Therefore, a multicenter, large-sample clinical trial is recommended to compare the modified APCS with existing screening tools, and to follow up participants to assess the long-term effectiveness of the combined screening tools in reducing CRC-related mortality and improving survival.

## Conclusion

In summary, in opportunistic screening of CRC, modified APCS combined with FOBT is used as a preliminary screening tool to stratify the risk of screening population to determine the priority and necessity of colonoscopy. This strategy can detect most advanced adenomas and reduce colonoscopies by a certain percentage. It is simple and convenient to operate, and is more suitable for large-scale promotion. Furthermore, tumor gross morphology, tumor differentiation degree, and nerve infiltration were independent risk factors for T1 CRC LNM.

## Supplementary Information

Supplementary Material 1.

Supplementary Material 2.

## Data Availability

Data is provided within the manuscript or supplementary information files.
